# Creation of Interpretable Summary Measures in Displaying Results from Mixed-effects Logit Models

**DOI:** 10.4172/2155-6180.1000304

**Published:** 2016-05-31

**Authors:** Xian Liu, Bradley E. Belsher, Daniel P. Evatt

**Affiliations:** 1 DoD Deployment Health Clinical Center, Defense Centers of Excellence for PH and TBI, 1335 East-West Highway, Silver Spring, Maryland, USA; 2 Department of Psychiatry, F. Edward Hebert School of Medicine, Uniformed Services University of the Health Sciences, Bethesda, Maryland, USA

**Keywords:** Binary longitudinal data, Conditional effect, Conditional odds ratio, Delta method, Mixed-effects logit model, Selection bias

## Abstract

The authors of this article developed new approaches to present analytic results from mixed-effects binary logit models in longitudinal data analysis. We first described basic specifications of mixed-effects logit models, the derivation of the fixed and the random effects, and nonlinear predictions of the response probability and the corresponding standard errors. Particular attention was paid to the interpretability of the conventional odds ratio in the longitudinal setting. The authors contended that without information on averaging of the random effects for two population subgroups of interest, the regression coefficient of an explanatory variable and its antilog in mixed-effects binary logit models are not interpretable. We recommended the computation of the conditional effect and the conditional odds ratio to aid in displaying a covariate's effect on the longitudinal binary response. An empirical illustration was provided to demonstrate how to create interpretable summary measures for aiding in the interpretation of the results from mixed-effects logit models when analyzing binary longitudinal data.

## Introduction

In longitudinal analysis, medical researchers frequently come across outcome data taking only two values. There are many examples of binary outcome data in medical and epidemiological studies. An older person with a serious health condition may be alive or dead at the end of a time interval following a medical treatment; a patient with depression experiences emission or remains diagnostically depressed after a psychosocial treatment; a drug-user either relapses to use drugs again or remains clean after being released from a rehabilitative center; and so on. When the distribution of the response variable is dichotomous in the longitudinal setting, the use of linear mixed models are no longer statistically efficient and can result in unrealistic predicted values of the response [1-5]. Additionally, the variance/covariance structure for the dichotomous response is not homogeneous longitudinally, thereby introducing additional specification problems. With such a data structure, mixed-effects logistic regression models, also referred to as mixed-effects logit models, are regularly applied for describing the time trend in the dichotomous outcome, as associated with theoretically relevant risk factors and confounders [1,3,4,6,7].

When interpreting the analytic results from mixed-effects binary logit models, one cannot solely rely on the estimated regression coefficients of explanatory variables and their antilog transforms (the so-called odd ratios). With incorporation of the individual-specific random effects, the covariate's effect on the response probability involves information on retransformation of the random components. Given such functional retransformation, the estimated regression coefficient from mixed-effect binary logit models may not be consistent with changes in the response probability with a unit change in the covariate. Therefore, in the analysis of binary longitudinal data researchers must evaluate the effects of explanatory variables by averaging over the distribution of the specified random components [2,6]. One perspective to accomplish this goal is to perform nonlinear predictions using values of the explanatory variables, the estimated regression coefficients, and the averaging of the random effects. In this perspective, a transformed linear function needs to be converted to predict the marginal probability, with normality of the random components in the logit function being appropriately retransformed to a nonnormal distribution [8.^9^]. Without such a retransformation process, the analytic results of mixed-effect binary logit models cannot be appropriately interpreted, even when true values of the regression coefficients are known.

In this article we attempt to go beyond existing work by introducing two new effect summary measures to aid in interpreting the results from mixed-effects logit models. First, we review the general specifications of mixed-effects logit models including computation of the predicted probability. Then, we describe the approximation method to estimate variance of the predicted probability. Next, we introduce the algorithms for two summary measures, the conditional effect on the probability and the conditional odds ratio, for displaying the effect of a specific risk factor in mixed-effects logit models. An empirical illustration is provided to display how to apply the new methods in longitudinal data analysis. In the summary section, we summarize merits and remaining issues in these methods.

## Mixed-effects Logistic Regression and Predicted Probability

Let *Y_ij_* denote the value of a dichotomous variable taking only two levels (for example, yes/no or 0/1) associated with subject *i* at time *j*, and Prob(Y_ij_ =1|***X***_ij_, ***b***_i_) be the probability of the subject taking value 1 at time *j* given covariate vector *X_ij_* and random efect vector ***b**_i_*. By adding the individual-level random effects to the classic logistic regression, the probability that *Y_ij_*=1 for person *i* at time *j* can be written by

(1) $$P_{ij}=\text{Pr}(Y_{ij}=1|X_{ij},b_{i})={\left[1+\exp(X_{ij}^{\prime }\beta +Z_{ij}^{\prime }b_{i})\right]}^{-1}\exp(X_{ij}^{\prime }\beta +Z_{ij}^{\prime }b_{i})={\left[1+\exp(X_{ij}^{\prime }\beta )\exp(Z_{ij}^{\prime }b_{i})\right]}^{-1}\exp(X_{ij}^{\prime }\beta )\exp(Z_{ij}^{\prime }b_{i}),$$

where *X_ij_* is the *M* × 1 covariate vector including a time variable or a set of time polynomials for subject *i* at time point *j*, *β* is an *M* × 1 vector of unknown population parameters with the first element being the intercept, *Z_ij_* is a known *n_i_* × *q* design matrix with the first column taking constant 1 if the intercept is assumed to be random across subjects, and *b_i_* is an *q* × 1 vector of the unknown subject effect with variance-covariance matrix ***G***. With the specification of *b_i_*, intra-individual correlation is addressed in mixed-effects logit models. By definition, the probability for *Y_ij_*=0 is. 1– *P_ij_* There are a variety of statistical approaches to derive efficient, consistent, and robust estimators of ***β***, ***b**_i_*, and ***G*** [10-17].

Equation (1) does not specify a term for within-subject random errors, based on the assumption that variations over individual-level random effects completely reflect within-subject variability in the response [1,8,18]. This assumption can sometimes be too restrictive as it implies perfect intra-persons correlation in longitudinal data assuming that uncertainty in the binary response at any time can be ignored. Overlooking sizable within-subject variability can result in tremendous bias in nonlinear predictions, thereby misspecifying the experiences generated by the stochastic longitudinal process [6,9].

If within-subject variability is included in mixed-effects logit models to address uncertainty given the model parameters, the probability *P_ij_*=1 for subject *i* at time *j* can be predicted empirically by

(2) $${\hat{P}}_{ij}|X_{ij},{\hat{b}}_{i}={\left\{1+\exp[-(X_{ij}^{\prime }\hat{\beta }+Z_{ij}^{\prime }{\hat{b}}_{i}+{\hat{\Delta }}_{ij})]\right\}}^{-1}={\left[1+\exp(-X_{ij}^{\prime }\hat{\beta })\exp(-Z_{ij}^{\prime }{\hat{b}}_{i})\exp(-{\hat{\Delta }}_{ij})\right]}^{-1},$$

where Δ̂*_ij_* is empirically defined as the second-order smearing estimate evaluated at (*β̂*, ***b̂***_i_), assumed to follow local normality. As the actual probability is unobservable, this within-subject random term can be approximated from the partial derivative of the log likelihood function with respect to ***β***, given by

$${\hat{\Delta }}_{ij}=\hat{\Delta }(y_{ij}-\mu_{ij})\approx {\frac{\partial 1(y_{ij}|\beta ,G,\varphi )}{\partial \beta }|}_{\hat{\beta },\hat{b}},$$

where μ_ij_ is the conditional mean. As a local approximation, this random term can be ignored only when intra-individual correlation is equal or close to unity implying that between-subjects variability can completely or predominantly capture the within-subject uncertainty.

If the within-subject random error is considered non-ignorable in the specification of a mixed-effects logit model, the conditional mean of the response probability *P_ij_* can be predicted by the following nonlinear function:

(3) $${\hat{P}}_{ij}|X_{ij},{\hat{b}}_{i}=\frac{1}{1+\exp\{-(X_{ij}^{\prime }\hat{\beta }+Z_{ij}^{\prime }{\hat{b}}_{i}+{\hat{\Delta }}_{ij})\}}={\left[1+\exp(-X_{ij}^{\prime }\hat{\beta })\exp(-Z_{ij}^{\prime }{\hat{b}}_{i})\exp(-{\hat{\Delta }}_{ij})\right]}^{-1}={\left[1+\exp(-X_{ij}^{\prime }\hat{\beta })(\frac{1}{{\hat{\Phi }}_{ij}})\right]}^{-1},$$

which has properties E(***b**_i_*)=**0**, cov(***b**_i_*)=***G***, and cov(***b**_i_*, *ε_ij_*)=**0**. In this mixed-effects logit model, the multiplicative random variable for subject *i* at time point *j*, denoted by **Φ**_ij_|***b***_i_, follows a multivariate lognormal distribution with expectation

(4) $$E(\Phi_{ij}|b_{i})=\exp(\frac{Z_{ij}{GZ}_{ij}^{\prime }+\sigma_{\varepsilon_{ij}}^{2}}{2}),$$

and variance

(5) $$\mathrm{var}(\Phi_{ij}|b_{i})=\exp[2(Z_{ij}{GZ}_{ij}^{\prime }+\sigma_{\varepsilon_{ij}}^{2})-\exp(Z_{ij}{GZ}_{ij}^{\prime }+\sigma_{\varepsilon_{ij}}^{2})].$$

If Φ̂_ij_ and ***X**_ij_* in Equation (3) are replaced with E(Φ_ij_) and ***X***_0_, respectively, Equation (3) predicts the marginal probability for a group of subjects with covariates evaluated at ***X***_0_. The individual probabilities within the group should be scattered randomly around the marginalized probability corresponding to variability of both between-subjects and within-subject random components. This relationship between the subject-specific and the marginal logit model is graphically and analytically demonstrated in Diggle *et al*. and Molenberghs and Verbeke [1,5]. Because the expected value of Φ̂_ij_ is greater than unity given a lognormal distribution, it is inappropriate to overlook this retransformed random variable in predicting a marginal probability unless $Z_{ij}{GZ}_{ij}^{\prime }=\sigma_{\varepsilon_{ij}}^{2}=0$.

## Approximation of Variance for the Predicted Probability

With the response probability being predicted, variance of the prediction needs to be approximated, as routinely applied in longitudinal modeling for nonlinear predictions [1,7,8,18]. Let *ℒ̂_ij_* be a random variable of the predicted logit for subject *i* at time point *j* with mean *η_ij_* ( $\eta_{ij}=X_{ij}^{\prime }\beta +\log\varphi_{ij}$, where log *φ_ij_* is the random term in the linear predictor) and variance var(*ℒ̂_ij_*), and P̂_ij_ = g^-1^(*ℒ̂*_ij_) is a transform of *ℒ̂_ij_*, as predicted by Equation (3). As conventionally defined, g is the logit link function and g^-1^ is its inverse function. For large samples, the first-order Taylor series expansion of g^-1^ (*ℒ̂_ij_*) yields approximation of mean, given by

(6) $$E[g^{-1}({\hat{L}}_{ij})]\approx g^{-1}(\eta_{ij}),$$

and the variance var(*P̂_ij_*)

(7) $$\mathrm{var}[g^{-1}({\hat{L}}_{ij})]\approx {\left[\frac{\partial g^{-1}({\hat{L}}_{ij})}{\partial {\hat{L}}_{ij}}|{\hat{L}}_{ij}=\eta_{ij}\right]}^{2}\mathrm{var}({\hat{L}}_{ij}).$$

In Equation (7), var⌊g^-1^(*ℒ̂_ij_*)⌋ is the approximate of variance of the predicted probability *P̂_ij_* for large samples. The calculation of the partial derivative in the equation can be based on a basic formula in calculus for the derivation of a ratio of two one-dimensional functions [4]. After some simplification, the partial derivative can be written in the formulation of the logit function, given by

(8) $$[\frac{\partial g^{-1}({\hat{L}}_{ij})}{\partial {\hat{L}}_{ij}}|{\hat{L}}_{ij}=\eta_{ij}]=\frac{\exp({\hat{L}}_{ij})}{{\left[1+\exp({\hat{L}}_{ij})\right]}^{2}}.$$

We might insert Equation (8) into (7), which yields the approximate of variance for *P̂_ij_*, given by

(9) $$\mathrm{var}[{\hat{P}}_{ij}]\approx {\left\{\frac{\exp({\hat{L}}_{ij})}{{\left[1+\exp({\hat{L}}_{ij})\right]}^{2}}\right\}}^{2}\mathrm{var}({\hat{L}}_{ij}),$$

where var(*ℒ̂_ij_*) may consist of two variance components – between-subjects variance and within-subject variance. While between-subjects variance component is generally estimated by the application of mixed-effects logit models, within-subject random errors can be approximated from the variance of the intercept with the covariates being rescaled to be centered at selected values.

## Conditional Effect of a Covariate on the Predicted Probability

In the construct of mixed-effect logit models, each subject has a unique random effect value on the log odds, and consequently, the regression coefficients are interpretable only within subjects or on the condition that two subjects have exactly the same value of the random effect [5]. As the random effect varies over subjects, the mean of the random effects also tends to change across different population subgroups with specific individual and environmental characteristics. Due to a lack of interpretability for the regression coefficients in mixed-effects logit models, the researcher can compute the discrete probability change with a one-unit increase in an independent variable [4,19], in which the averaging of the random effects must be taken into account. This method compares the nonlinear predictions between two population subgroups or given two values of independent variables while adjusting for the confounding effects. As the change in the predicted probability is conditional on the value of covariates, the estimated regression coefficients, and the average of the random effects associated with the two population subgroups, we refer to this scale-dependent effect as the *conditional effect*.

For analytic convenience, consider the effect of a medical treatment factor at a given time point. We define the treatment factor as *X_m_* that takes value 0 (control) or 1 (treatment). Let (P̂_ij_|X_m_ = 0, ***X̄***_r_, Φ̄_0_) be the marginalized estimate of the response probability for the control group when all other covariates are scaled as sample means, where ***X̄****_r_* is a vector containing sample means of the covariates other than the treatment factor and Φ̄_0_ is the average of the random effects among those in the control group. Likewise, let (P̂_ij_|X_m_ = 1, ***X̄***_r_, Φ̄_m_) be another predicted marginal mean for those receiving treatment when other covariates are fixed at sample means where Φ̄_m_ is the average of the random effects for those receiving treatment. It follows that the difference between (P̂_ij_|X_m_ = 1, ***X̄***_r_, Φ̄_m_) and (P̂_ij_|X_m_ = 0, ***X̄***_r_, Φ̄_0_) is the conditional effect of the treatment variable *X_m_* on the response probability, marginalized at sample means, denoted by ΔP̂_m_. The equation is

(10) ΔP^m=exp(β^m+X¯r′β^r)Φ¯m1+exp(β^m+X¯r′β^r)Φ¯m−exp(X¯r′β^r)Φ¯01+exp(X¯r′β^r)Φ¯0,

Where β̂_m_ is the estimated regression coefficient of the dichotomous treatment variable and *β̂* is the vector of the estimated regression coefficient for the other covariates. Equation (10) can be readily extended to computing the conditional effect of a continuous independent variable on the response probability. This discrete probability change approach differs conceptually from the conventional marginal effect described in the literature of econometrics reflecting an instantaneous rate change without bound in value [20]. In the application of mixed-effects logit models, we strongly recommend the use of the discrete conditional effect because it is consistent with the traditional perspective to interpret the effect of a covariate and accommodates the specification of qualitative independent variables taking more than two values. Theoretically, the conditional effect on the probability scale is scale-dependent, and therefore, it is sensitive to change in the value of the covariate. The logistic function, however, approximates a straight line except at the two ends, and therefore, the conditional effect does not tend to vary considerably over changes in the covariate's scale within the zones where most cases are located.

A statistically significant effect of a covariate on the logit scale does not necessarily translate into a statistically significant effect on the probability scale. While the significance statistic on the logit scale only tests the significance of the fixed effect, a significance test on the conditional effect at the probability scale accounts for variability of all the random components. Therefore, the significance tests on these two different scales, logit and probability, need to be performed separately. Specifically, statistical significance of the conditional effect can be tested by the Wald chi-square statistic, denoted by $\chi_{W,m}^{2}$ [21,22].

Let *P̂*_0_ represent (P̂|X_m_ =0, ***X̄***_r_, Φ̄_0_) and *P̂_m_* stand for (P̂|X_m_ =1, ***X̄***_r_, Φ̄_m_). The following equation of the Wald chi-square statistic is then defined from the delta method:

(11) $$\chi_{W,m}^{2}\approx \frac{{\left({\hat{P}}_{m}-{\hat{P}}_{0}\right)}^{2}}{\mathrm{var}({\hat{P}}_{0})+\mathrm{var}({\hat{P}}_{m})-2\mathrm{cov}({\hat{P}}_{0}{\hat{P}}_{m})}.$$

As predicted from the same parameter estimates where all but one covariate has exactly the same values, *P̂*_0_ and *P̂_m_* are essentially subject to the same probability distribution. Therefore, the two random variables *P*_0_ and *P_m_* should be very closely correlated. For analytic convenience, the two nonlinear predictions, *P̂*_0_ and *P̂_m_*, can be assumed to have perfect correlation [22]. Consequently, Equation (11) can be simplified as

(12) $$\chi_{W,m}\approx \frac{\left({\hat{P}}_{m}{\hat{P}}_{0}\right)}{\mathrm{var}({\hat{P}}_{0})+\mathrm{var}({\hat{P}}_{m})-2\sqrt{\mathrm{var}({\hat{P}}_{0})\mathrm{var}({\hat{P}}_{m})}}$$

This Wald statistic is distributed asymptotically as chi-square with one degree of freedom under the null hypothesis that (*P̂_m_* – *P̂*_0_)= 0. If the assumption of perfect correlation between *P̂*_0_ and *P̂_m_* is considered invalid with specification of the random effects, Equation (11) can be applied by using an empirical covariance score.

## Conditional Odds Ratio of a Covariate

With specification of the random effects, the antilog transform of the regression coefficient, conventionally interpreted as the odds ratio, becomes hard to interpret. Given the same example as above, the odds ratio for the treatment factor *X_m_* at time point *j*, is the ratio of the odds of *Y*=1 for those receiving treatment to the odds for those in the control group. In the longitudinal setting, the odds ratio can be written as

$${\text{OR}}_{m}|t_{j},{\bar{X}}_{r}=\frac{\text{Prob}[Y_{j}=1|X_{m}=1,{\bar{X}}_{r},{\bar{\varphi }}_{mj}]}{\text{Prob}[Y_{j}=0|X_{m}=1,{\bar{X}}_{r},{\bar{\varphi }}_{mj}]}÷\frac{\text{Prob}[Y_{j}=1|X_{m}=0,{\bar{X}}_{r},{\bar{\varphi }}_{0j}]}{\text{Prob}[Y_{j}=0|X_{m}=0,{\bar{X}}_{r},{\bar{\varphi }}_{0j}]}=\frac{\text{Prob}[Y_{j}=1|X_{m}=1,{\bar{X}}_{r},{\bar{\varphi }}_{mj}]\text{Prob}[Y_{j}=0||X_{m}=0,{\bar{X}}_{r},{\bar{\varphi }}_{0j}]}{\text{Prob}[Y_{j}=0|X_{m}=1,{\bar{X}}_{r},{\bar{\varphi }}_{mj}]\text{Prob}[Y_{j}=1||X_{m}=0,{\bar{X}}_{r},{\bar{\varphi }}_{0j}]}.$$

Clearly, in computing the odds ratio in mixed-effects logit models, the averaging of the random effects needs to be taken into account.

After some mathematical simplification, the odds ratio for the treatment factor at time point *j* can be computed in the construct of mixed-effects logit models, given by

(13) $${\text{OR}}_{m}=\frac{\exp({\hat{\beta }}_{m}+{\bar{X}}_{rj}^{\prime }{\hat{\beta }}_{r}){\bar{\Phi }}_{mj}}{\exp({\bar{X}}_{rj}^{\prime }{\hat{\beta }}_{r}){\bar{\Phi }}_{0j}}=\exp({\hat{\beta }}_{m}+{\bar{X}}_{rj}^{\prime }{\hat{\beta }}_{r}-{\bar{X}}_{rj}^{\prime }{\hat{\beta }}_{r})(\frac{{\bar{\Phi }}_{mj}}{{\bar{\Phi }}_{0j}})=\exp({\hat{\beta }}_{m})(\frac{{\bar{\Phi }}_{mj}}{{\bar{\Phi }}_{0j}}).$$

Equation (13) can be well extended to the odds ratio with a one-unit change in a continuous covariate. This equation clearly demonstrates that exponentiation of β̂_m_ does not result in an odds ratio in longitudinal analysis without information on the averaging of the random effects for two population subgroups. Given dependence on the averaging of the random effects, the odds ratio specified in Equation (13) is referred to as the *conditional odds ratio*.

The variance of the conditional odds ratio for the treatment factor can be approximated by using the delta method. Let g̃(*β̂_m_*) be a single-valued function linking *β̂_m_* to the odds ratio of *X_m_*. Then, from the Taylor series expansion of the function g̃(*β̂_m_*), variance of the odds ratio can be approximated by

(14) $$\mathrm{var}({\text{OR}}_{m})=\mathrm{var}[\tilde{g}({\hat{\beta }}_{m})]\approx {\left[{\tilde{g}}^{\prime }({\hat{\beta }}_{m})\right]}^{2}\mathrm{var}({\hat{\beta }}_{m}).$$

Given the third equality of Equation (13), Equation (14) can expand to

(15) $$\mathrm{var}({\text{OR}}_{m})={\left\{{\left[\exp({\hat{\beta }}_{m})\frac{{\bar{\Phi }}_{m}}{{\bar{\Phi }}_{0}}\right]}^{\prime }\right\}}^{2}\mathrm{var}({\hat{\beta }}_{m})={\left\{\exp({\hat{\beta }}_{m})[\frac{{\bar{\Phi }}_{0}-{\bar{\Phi }}_{m}}{{\bar{\Phi }}_{0}^{2}}]+\exp({\hat{\beta }}_{m})[\frac{{\bar{\Phi }}_{m}}{{\bar{\Phi }}_{0}}]\right\}}^{2}\mathrm{var}({\hat{\beta }}_{m}),={\left\{\exp({\hat{\beta }}_{m})[\frac{{\bar{\Phi }}_{0}-{\bar{\Phi }}_{m}+{\bar{\Phi }}_{m}{\bar{\Phi }}_{0}}{{\bar{\Phi }}_{0}^{2}}]\right\}}^{2}\mathrm{var}({\hat{\beta }}_{m}).$$

The standard error of the conditional odds ratio is the square root of the conditional odds ratio variance, based on which the confidence interval of the conditional odds ratio can be easily computed. Given nonlinearity of the probability distribution after retransformation, however, this confidence interval is not symmetric.

In displaying the results from mixed-effect logit models, the conditional odds ratio does not necessarily reflect the magnitude of the difference in the probability between two population subgroups of interest. For example, a ratio of 0.4 over 0.2 is two, and the ratio of 0.004 over 0.002 is also two, in which the information about the difference between the absolute probabilities is immensely overlooked. Correspondingly, the variance of an odds ratio estimate in logistic regression reflects variations in a ratio, rather than the variability in the difference between two predicted probabilities. In the application of mixed-effects logit models, therefore, significance test on the effect of a risk factor on the response variable cannot rely on the variance estimator for the conditional odds ratio. It is scientifically sounder to display the conditional effect of a risk factor on the probability when presenting the results from mixed-effects logit regression models.

## Illustration

The data presented here is solely for the purposes of illustrating interpretation of the results from mixed-effects logit models. Data used for this illustration came from a partially empirical and partially simulated dataset, derived from a two-parallel arm randomized, controlled effectiveness trial evaluating a care management intervention for treating Posttraumatic Stress Disorder (PTSD) and Depression in primary care. A detailed description of the study design, procedures, measures, and results is available elsewhere [23,24]. Briefly participants were recruited directly from eighteen primary care medical clinics at six large US military treatment facilities. Participants who met criteria for probable PTSD and/or depression were identified by and referred to the study through routine PTSD and depression screening in military primary care and at the discretion of the primary care provider and the patient. Thus, the sample was representative of patients who were presenting to military primary care with PTSD and/or depression symptoms and accepted a referral to receive management of their condition within primary care. The final sample of 666 eligible and consented patients (332 active interventions; 334 cares as usual) was drawn from 2,592 patients who were referred to care management during the project period. PTSD and depression outcomes were measured longitudinally across four time-points in one year (Baseline, 3-Months, 6-Months, 12-Months).

In this illustration, we analyzed the impact of the active intervention on the pattern of change over time in diagnostic status. Participants with eleven symptoms of PTSD and/or depression were categorized as diagnostic, while those patients below threshold cutoff levels for both PTSD and depression were categorized as diagnosis-free. Thus, the outcome diagnostic status variable was dichotomous, with 1=diagnostic and 0=diagnostic-free. Operationally, we analyzed the probability of being diagnostic at four time points, defined as Pr(Y_*ij*=_) 1where *i*=1, …., *N*, *j*=1, 2, 3, 4. Correspondingly, the complement of the diagnostic probability, namely 1-Pr (Y_*ij*=_) 1, was defined as the diagnostic-free probability. Of the explanatory variables, time was the primary predictor to describe trends of diagnosis over time, with the number of months from baseline being measured (0, 3, 6, 12 months). Another main predictor is treatment arm, for which we defined 1=active intervention and 0=care as usual intervention. Three control variables were included in regression analysis to adjust for confounding effects: gender (1=women and 0=men), age at baseline, and education at baseline. To adjust for confounding effects effectively, we rescaled values of these controls to be centered at sample means at each time point.

As the response data were binary, the random-intercept binary logit model was applied, and accordingly, we assumed the effects of the explanatory variables on the logit to be fixed over time. We applied the SAS PROC NLMIXED procedure (SAS Institute Inc., Cary, NC) to derive parameter estimates, both the fixed and the random [25]. According to preliminary data analysis, adaptive Gaussian quadrature was used for the approximation of the integral of the likelihood over the random intercept. In terms of deriving robust random effect estimates and the model fit statistic, the advantage of adaptive Gaussian quadrature over other approximation methods has been well documented [5,16]. With use of the random intercept logit model, we specified time as a continuous variable. After a thorough examination, we chose to use the quadratic polynomial time function, the combination of time and time × time components, to describe trends of diagnosis. As a routine practice, we rescaled the time variable *T* as a centered covariate to reduce numeric instability and collinearity [4].

Table 1 displayed the analytic results of the mixed-effects logit model with covariates, except the treatment factor, being centered at sample means at time six (the third time point). The regression coefficients of time, time × treatment, and time × time were each statistically significant at *α*=0.05 reflecting high quality of these estimates. While the main effect of the treatment factor was not shown to be statistically significant (*β*=-0.166, se=0.273, *t*-value=-0.61, *p*-value > 0.05), we consider the fixed effect of the active intervention to be statistically meaningful given its strong interactive association with time. It may be indicated again that exponentiation of the treatment fixed effect did not convert to an effective odds ratio estimate without averaging of the random effects for the two treatment groups. The between-subjects random effect on the intercept was statistically significant, suggesting very high intra-individual correlation in the active intervention longitudinal data. Of the three control variables, age had a positive, statistically significant effect on the log odds of the diagnostic probability, as expected. Males were expected to have positive effects on the log odds, and highly educated persons to have negative effects on the log odds; however, the regression coefficients of those two controls were not statistically significant. The fixed effects of covariates did not yield a robust and consistent estimator for interpretation of the results from mixed-effects logit models. In longitudinal analysis on binary data, nonlinear predictions and the computation of conditional effects for covariates are essential.

The predicted diagnostic probability at each time point and its variance can be estimated by applying Equations (3) and (9), respectively. Table 2 presents the predicted diagnostic probability across four time points (0, 3, 6, and 12 months) for both the control and the treatment groups. For both intervention groups, the diagnostic probability is shown to decline steadily over time. For the care as usual group, the probability declined from 0.952 at baseline to 0.786 at month twelve, a 17.4 percent reduction in diagnostic probability; for the active intervention group, reduction during the same observation period was even greater, a 25% decline (0.961 at baseline to 0.720 at month twelve). Additionally, both sets of the diagnostic probability over time were associated with very low values of variance, indicating high quality of these nonlinear predictions.

Table 2 displayed a delayed effect of the active intervention. At the first two time points, time zero and time three, the difference in the diagnostic probability was negligible, though statistically significant. The conditional effects of the active intervention at those two months were 0.009 and 0.004, respectively. At month 6, the diagnostic probability for the active intervention group started to decline more sharply than for the care as usual group, 0.720 versus 0.810. At the last time point, month twelve, the difference in the diagnostic probability between the two intervention groups was as substantial as 7 percent, statistically significant with a high value of the Wald-statistic (chisq=51.09 with one degree of freedom). The conditional odds ratios also showed the delayed effect of the active intervention; nevertheless, as a relative score, the odds ratio failed to reflect the change over time in the effect of intervention accurately. For example, the conditional odds ratio at baseline was as high as 1.25, statistically significant, while the absolute difference in the diagnostic probability between the treatment groups was less than 0.01.

**Figure 1:**
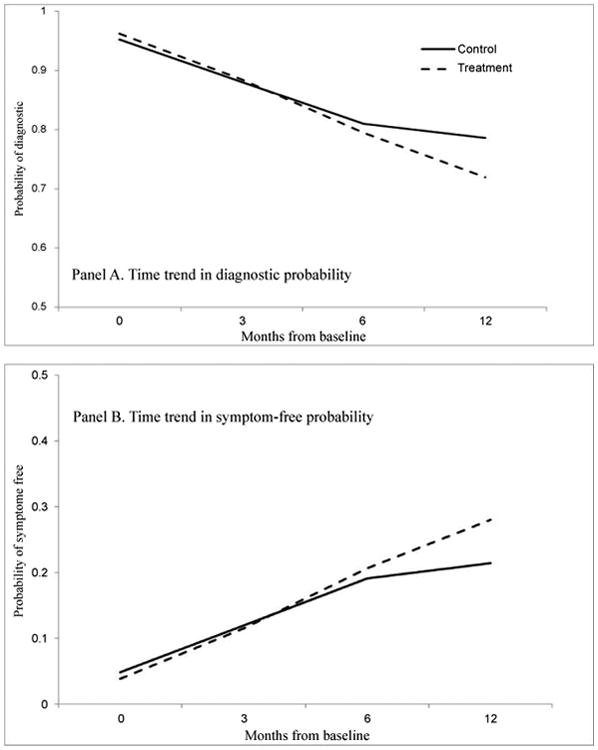
Pattern of change over time in diagnostic and diagnosis-free probabilities: treatment and control groups (*N*=666).

Figure 1 plots longitudinal trajectories of the diagnostic probability and its complement, the diagnosis-free probability. The solid line represented the trajectory of those in the care as usual group, while the dotted curve for patients receiving the active intervention. Panel A demonstrated a trend of sustained reduction in the diagnostic probability and its differences between the two intervention groups. At the first two time points, the two trajectories displayed a sharply declining pattern at almost the same pace; the two lines started to separate at month 6 and eventually leading to a considerable separation at month twelve. In Panel B, the pattern of change over time in the diagnosis-free probability was displayed. As a complement to the diagnostic, the diagnosis-free trajectory displayed the same rate of change to that of Panel A, though with the opposite direction, with a wide separation between the two curves at the last time point. The trajectories of the diagnostic and the diagnosis-free probabilities each have their own focuses and implications, and therefore, the researcher might want to select which trajectory curve should be presented according to one's research interests.

## Discussion

We argued in this article that one should not attempt to interpret the analytic results of mixed-effects logit models by means of the regression coefficients or their antilog transforms. The reason was that with the specification of the individual-specific random effects, a normally distributed random component on the logit function must be converted to a lognormally distributed multiplier in nonlinear predictions of the response probability. Given the property of a lognormal distribution, the expectation of this multiplier was greater than unity in nonlinear predictions, and this expectation tended to take different values over various population subgroups due to the presence of the variance function in generalized linear mixed models. Additionally, test results could be tremendously biased from ignoring retransformation of the random components because the variance of nonlinear predictions would be considerably underestimated. In this study, we attempted to provide new summary measures to correctly interpret the analytic results from mixed-effects logit models. In our perspective, both between-subjects and within-subject variability and its retransformation were taken into account in nonlinear predictions and the computation of the covariate's effect.

We would also like to emphasize that when analyzing longitudinal binary data, one needs to examine the effects of explanatory variables on the response probability and the effects on the logit separately. When the random effects are considered, both types of covariate effects are time-dependent, conditional on the covariates' values, the regression coefficients, and the averaging of the random effects. Sometimes, those two types of covariate effects, both point and variance estimates, can differ considerably, in turn generating different test results. Compared to the odds ratio statistic, the predicted probability and the conditional effect of a risk factor reflect a longitudinal trend and its difference between two associated population subgroups in a straightforward fashion. Therefore, these two summary measures provide more useful information with policy implications than the odds ratio statistic.

## Figures and Tables

**Table 1:** Analytic results and summary measures for the mixed-effects logit model on diagnostic status (*N*=666; *df*=665).

Explanatory variable and effect measure	Regression coefficient	Standard error	*t* value	*p* value> |t|
Fixed effects				
Intercept	2.474***	0.250	9.88	<0.01
Time (centered at month six)	-0.211***	0.028	-7.45	<0.01
Treatment	-0.166	0.273	-0.61	0.54
Time (centered) × treatment	-0.075**	0.037	-2.02	0.04
Time × time (centered)	0.028***	0.005	5.49	<0.01
Age (centered at month six)	0.099***	0.023	4.38	<0.01
Male (centered at month six)	0.307	0.345	0.89	0.37
Educ. (centered at month six)	-0.093	0.099	-0.94	0.35
Random Effects:				
Intercept	2.513***	0.214	11.76	<0.01
-2 log likelihood	1756.90			

*** *p*-value <0.01;

** 0.01<*p*-value <0.05.

Note: Randomness of the intercept is parameterized by the standard error of the random effects.

**Table 2:** Predicted probabilities of diagnostic for treatment and control groups and three treatment effect summary measures (*N*=666).

Predicted probability of diagnostic and effect of treatment	Month since baseline time			
	Baseline time	month three	month six	month twelve
Probability for control group	0.952 (var <0.001)	0.881 (var <0.001)	0.810 (var <0.001)	0.786 (var <0.001)
Probability for treatment group	0.961 (var <0.001)	0.885 (var <0.001)	0.794 (var <0.010)	0.720 (var <0.001)
Conditional effect of treatment	0.009 (chisq >100)	0.004 (chisq >100)	-0.016 (chisq=98.0)	-0.066 (chisq=51.1)
Conditional odds ratio of treatment	1.250 (var=0.007)	1.039 (var=0.007)	0.907 (var=0.007)	0.699 (var=0.082)
